# *Bartonella* Infection in Immunocompromised Hosts: Immunology of Vascular Infection and Vasoproliferation

**DOI:** 10.1155/2012/612809

**Published:** 2011-11-17

**Authors:** Mosepele Mosepele, Dana Mazo, Jennifer Cohn

**Affiliations:** Department of Medicine, Hospital of the University of Pennsylvania, 100 Centrex, Philadelphia, PA 19104, USA

## Abstract

Most infections by genus *Bartonella* in immunocompromised patients are caused by *B. henselae* and *B. quintana*. Unlike immunocompetent hosts who usually develop milder diseases such as cat scratch disease and trench fever, immunocompromised patients, including those living with HIV/AIDS and posttransplant patients, are more likely to develop different and severe life-threatening disease. This paper will discuss *Bartonella's* manifestations in immunosuppressed patients and will examine *Bartonella's* interaction with the immune system including its mechanisms of establishing infection and immune escape. Gaps in current understanding of the immunology of *Bartonella* infection in immunocompromised hosts will be highlighted.

## 1. Introduction

In this paper, we present a summary of the basic characteristics of *Bartonella* species which are important etiologic agents in human disease. The discussion focuses on a review of what is known of the immunology of *Bartonella*, particularly in immunocompromised hosts. 

## 2. Classification

*Bartonella* are fastidious hemotropic facultative intracellular Gram-negative bacteria of the alpha-2 subgroup of Proteobacteria [1, 2]. Of the 24 species that comprise the genus *Bartonella, B. bacilliformis, B. henselae, *and* B. quintana *infections have been most commonly implicated in human disease and *B. henselae* and *B. quintana* account for the majority of morbidity and mortality among immunocompromised individuals [3–5]. 

## 3. *Bartonella* Epidemiology

Each of the major *Bartonella* species implicated in human disease has unique epidemiology. While the vast majority of *Bartonella* species have an identified zoonotic reservoir such as domesticated animals and farm animals, the animal reservoir for both *B. quintana* and *B. bacilliformis* have not yet been identified. While *B. bacilliformis* is transmitted by the sand fly and the major vector for *B quintana *is the human body louse, *B quintana *has also been found in rodent fleas, including rat fleas, and thus a potential for a rodent vector has been hypothesized [6, 7]. 

*B. henselae* occurs throughout the world. It is a zoonosis transmitted from the natural reservoir, cats (*Felis catus*), usually via cat scratch or bite and less commonly by a vector such as cat fleas (*Ctenocephalides felis*) or ticks [8]. Cross-sectional studies have reported as much as 81% seropositivity point prevalence among cats in different regions of the world. Bacteremia is more prevalent in cats that are less than 1 year old [2]. Humans and dogs are accidental hosts.

Although *B. quintana* occurs worldwide, with improved sanitation and living conditions, disease caused by *B. quintana* has dramatically decreased and now occurs primarily in small epidemics in conditions characterized by crowding and poor sanitation. 

Humans are likely the natural reservoir for *B. quintana* which is spread by human body lice (*Pediculus humanis corporis*) [8]. Infected body lice excreta on human skin induce pruritis, and skin breakdown that follows scratching allows for *B. quintana's* pathway of invasion [9]. Wild rodents have also been identified as potential reservoirs [6, 7].

*B. bacilliformis* is endemic in mountainous areas of Peru, Columbia, and Ecuador [10]. It is transmitted by the *Lutzomyia* sandfly, but the animal reservoir has yet to be identified. 

## 4. *Bartonella* and the Human Immune System

### 4.1. Establishing Infection: Evasion of the Immune System

Despite extensive knowledge about the disease syndromes caused by *Bartonella* species in humans, little is known about the humoral and cellular immune mechanisms involved in establishment and persistence of *Bartonella* infection in humans. Experimental studies using *B. tribocorum* inoculation onto an immune-competent rat model have shown that the brief bacteremia after inoculation is followed by a 3–5-day bacteremia-free window before resurgence of persistent intraerythrocytic bacteremia that lasts for 8–11 weeks [11]. This model highlights possible existence of a sanctuary site where the initial infection gets established before persistent intraerythrocytic infection manifests. So far, three such potential sanctuary sites include (a) extracellular matrix where high local bacterial replication may be achieved following initial infection [11], (b) bone-marrow stem cells such as erythroid progenitor cells where CD34+ cells in the bone marrow may be infected followed by release of infected erythrocytes into the blood stream [12] or endothelial progenitor cells which may also get infected in the bone marrow prior to their mobilization into circulation for endothelial repair [13], and, finally, (c) mature endothelial cells [14].

**Bartonella Has Several Characteristics That Lead to Immune Evasion**

*Bartonella* lipopolysaccaride (LPS) is composed of a unique combination of Lipid A and long-chain fatty acids and likely contributes to the bacteria's ability to evade the immune system [15]. Empiric data have shown that *Bartonella* initially evades the innate immune system because its surface molecules are not recognized by TLR-4 on dendritic cells or macrophages, [16], thereby allowing for establishment of what might lead to persistent infection. The LPS does not induce tumor necrosis factor alpha and has reduced stimulation of Toll-like receptor 2 (TLR2) and thus reduced endotoxicity [17, 18]. The LPS of *B. quintana* may even antagonize the Toll-like receptor 4 (TLR4) [19], which is a key component of innate immunity. Popa et al. and Matera et al. demonstrated that LPS from *B. quintana *resulted in the downregulation in human monocytes of nearly all cytokines normally produced by TLR4 in response to LPS [19, 20] and *B. quintana's *antagonism of TLR4 may be responsible for the absence of symptoms normally associated with bacteremia with Gram-negative organisms, such as septic shock.

Furthermore, *B. henselae* can avoid lysosomal fusion and acidification after the bacteria invades phagocytes such as endothelial cells and macrophages. For instance, *B. henselae* containing vacuoles (BCVs) had delayed development or complete lack of typical endocytic markers [21]. When the BCV contained inert or killed *B. henselae*, the endocytic markers were in normal ranges. Such Dendritic cells within cat scratch disease (CSD) granuloma in immune-competent humans that are chronically infected with Bartonella are also able to limit the infection to the same lymph node region via a localized B-cell-mediated humoral immune activation that is regulated by dendritic cells [22]. On the contrary, immunocompromised persons develop bacteremia, and distant seeding may occur, hence recovery of *Bartonella* in cardiac valves, other vasoproliferative lesions (bacillary angiomatosis or peliosis). Exactly how local bacterial control is lost in immunocompromised subjects is unclear.

### 4.2. Bacterial Persistence: Pro- and Anti-Inflammatory Response

Historically, clinical and in vitro studies implicated a Th1 immune response to *Bartonella* infection. The old *Bartonella* skin test involved an intradermal injection of suppurative material derived from lymph nodes from cat scratch disease patients and was interpreted as positive if their skin developed a large erythematous area within 48 hrs. This test is a classic delayed type hypersensitivity reaction, mediated through the Th1 immune response. In vitro studies have also supported the use of a Th1 response with mice splenocytes showing increased production of the Th1 cytokines IFN*γ* and IL-12 in response to *B. henselea* when compared with controls [23]. The role of innate immunity is also demonstrated through the repeated evidence of elevated IL-8 in response to *B. henselae* [24].

More recent studies have focused on the role of cytokines in acute and chronic *Bartonella* infection. Acute infection in immune-competent subjects with CSD have revealed upregulation of proinflammatory (IL-2, IL-6) and anti-inflammatory (IL-10) cytokines [25]. On the contrary, low CD4 counts (a marker of immune-compromise) have been associated with elevated IL-10 levels during acute infection with *Bartonella*, a cytokine milieu that may allow an acute infection to persist especially given the anti-inflammatory nature of IL-10 [16, 25, 26]. Chronic infection is associated with elevated levels of Interferon-alfa and IL-4 in both animal [16, 27] and human studies of the bacillary angiomatosis [16], but not in immune-competent subjects with CSD [25]. Thus, far, empiric data regarding *Bartonella* infection persistence and role in vasoproliferation is better understood from cytokine profiles, and less so from the traditional roles of humoral or cellular immunity.

Most of the research in infection involving immunocompromised hosts has focused on the establishment of bacterial stimulated angiogenesis. Understanding of the immune defense of *Bartonella* species in immunocompromised patients has been hampered by the lack of good animal model [28]. Since the response in the competent immune system involves Th1 and innate immunity through macrophages, a logical inference can be made that HIV-positive and other patients deficient in these immunological areas would have difficulty limiting the infection and thus would develop systemic manifestations. This deduction, however, has not yet been demonstrated as the mechanism by which HIV facilitates the spread of *Bartonella*. Chiaraviglio et al. described a murine model for chronic *Bartonella* infection using *B. taylorii *in SCID/Beige mice (B-, T-, NK-cell deficient) [29]. The group described that this model approximated human bacillary peliosis and splenitis through *Bartonella* growing in extracellular aggregates in the spleen and liver. As the model is still relatively new, they did not present any further characterization of the immune response. However, it is clear that intraerythrocytic persistence is characteristic of infections in immune-competent hosts while endothelial/periendothelial persistence leading to vasoproliferation is characteristic of an infection in immune-compromised hosts [29].

## 5. Pathophysiology of *Bartonella * Disease/Lesions in Immunocompromised Hosts

### 5.1. Angiogenesis

Bacillary angiomatosis is the most common sequelae of *B. quintana* and *B. henselae* infections in patients with cell-mediated immunodeficiency such as HIV and posttransplant patients on immunosuppressive therapy [9, 23, 30].* B. henselae* and *B. quintana* induce their characteristic vasoproliferative lesion, bacillary angiomatosis, or peliosis, in immunosuppressed patients, by direct and indirect effects on endothelial cells.

Whereas most pathogenic bacteria achieve tissue destruction and wider dissemination by inducing apoptosis on host cells, *B. henselae* and *B. quintana* infections persist within periendothelial extracellular matrix resulting in sustained, localized bacterial replication within collagen tissue [29]. This localized bacterial replication facilitates an antiapoptotic state in endothelial cells by secreting effector proteins (BepA and BepA2) which bind to the endothelial membrane receptor. The ensuing transmembrane signal transduction results in high cytoplasmic cAMP levels. The high cAMP levels upregulate cAMP responsive genes and induce an antiapoptotic state in the endothelial cells, resulting in their proliferation [31]. In addition, Schmid et al. in the same experiment showed that BepA can inhibit cytotoxic T-cell-mediated apoptosis of endothelial cells infected by *B. henselae* and *B. quintana*. A further analysis of the antiapoptotic mechanisms of some *B. henselae* strains isolated from HIV-infected patients revealed that an anti-apoptotic state may be effected through *B. henselae* strain-specific upregulation of antiapoptotic or downregulation of proapoptotic factors that work through the mitochondrial intrinsic apoptotic pathway [32]. As noted earlier, Interferon-alfa and IL-4 in chronic *Bartonella* infections may play a role, albeit unclear, in the development of bacillary angiomatosis given that elevated levels have been reported both animal [27] and human studies of the bacillary angiomatsois [25], but not in immune-competent subjects with CSD [25].

NF-*κ*B is also upregulated by *Bartonella*, resulting in increased leukocyte rolling and adhesion and causes a proinflammatory environment [12]. Additional research is needed to explore this link between some *B. henselae* strains that infect HIV patients and vasoproliferation and inflammation. Such work may shed more light into pathogenicity of *B. henselae* and host susceptibility factors in HIV-positive patients. 

## 6. Immunocompromise Induced by *Bartonella * Infections

*B. bacilliformis* itself induced an immunocompromised state. Although *B. bacilliformis* is not widespread in distribution and has not been described as a cause of bacillary angiomatosis or hepatic peliosis in immunocompromised patients, it is notable that the acute bacteremic phase of *B. bacilliformis* causes significant immunosuppression by overloading the reticuloendothelial system. These patients are predisposed to infections such as salmonella and tuberculosis [28]. 

## 7. Clinical Features of *Bartonella * Infections in Immunocompromised Hosts

*Bartonella* infections caused by *B. henselae* or *B. quintana* in the immunocompromised host may be characterized by fever of unknown origin, culture-negative endocarditis, osteomyelitis, or angioproliferative lesions that may affect virtually any organ system, but have a predilection for the skin, liver, and spleen. 

### 7.1. Clinical Features of *Bartonella* Infections in HIV-Infected Patients

Bacillary angiomatosis lesions and fever are the most common manifestation of *B. henselae* or *B. quintana* infection in HIV/AIDS patients [3, 9]. The lesions appear as cutaneous nodular vascular lesions but may also be found in a variety of organs including the GI-tract where they may cause hematemesis [33–35], genitourinary system [36], and other organs including heart, spleen, bones, and central nervous system. The differential diagnosis for bacillary angiomatosis includes Kaposi's sarcoma, angiosarcoma, and pyogenic granuloma. 

Similar vascular lesions are seen in both bacillary peliosis and splenitis. Patients present with hepato- and/or splenomegaly as blood filled cysts proliferate in these organs. 

Confirming the diagnosis can be challenging as serology has a 50–95% sensitivity and demonstrates cross-reaction between *Bartonella*, *Coxiella,* and *Chlamydia* species. Results from tissue culture are not always positive and take a long time given the fastidious nature of the bacterium. PCR is favored as a faster, sensitive, and specific diagnostic test [37, 38]. 

### 7.2. Clinical Features of *Bartonella* Infections in Transplant Patients

Persistent fever following close contact with cats is the most common presenting symptom among posttransplant patients who are infected with *B. henselae* [39]. *B. henselae* infection was complicated by acute graft rejection in 2 renal transplant patients [40]. It is unclear if this rejection was a random occurrence or if it is associated with some unknown effect of *B. henselae* on decreasing the donor tolerance of the graft or enhancing graft antigenicity. In liver transplant patients, *B. henselae* can present with hepatic masses [41], hepatic granulomas [42, 43], or disseminated disease [44].

### 7.3. Clinical Features of *Bartonella* Infection in Homeless Patients with Chronic Alcoholism

Subacute, culture-negative endocarditis is the most common clinical presentation of *B. quintana* (less so *B. henselae*) infection among homeless patients with chronic alcoholism [4, 45, 46]. Risk factors for *Bartonella* endocarditis include preexisting valvular lesions and exposure to human body lice. 

*B. quintana* may be cultured from the blood, but given the prolonged time required for culture, other diagnostic tests are preferred. An indirect immunofluorescent IgG antibody test directed against *Bartonella* species with titer of 1 : 800 or higher has a 95% positive predictive value for diagnosing *Bartonella* endocarditis [47]. Diagnosis is usually confirmed by performing PCR testing on plasma or tissue samples following DNA extraction [6].

## 8. Conclusion

*Bartonella* species are important zoonotic agents of human disease. Immunocompromised hosts are more susceptible to infection from *B. henselae* or *B. quintana* which results in a more severe clinical picture as compared with the normal host. More recent work explores role of cytokines and chemokines in subverting the immune system, and subsequently a persistent infection characterized by vasoproliferation in immunocompromised humans. The mechanisms of establishing infection and immune evasion in immunocompromised hosts are still incompletely understood and are areas where further studies are needed. 
